# Long-Term Kidney and Patient Survival after Deceased Donor Transplantation in Two Brothers with Alport Syndrome from the Same Donor

**DOI:** 10.2478/bjmg-2026-0002

**Published:** 2026-10-14

**Authors:** G Severova, I Hamamdzieva, P Dzekova-Vidimliski, L Trajceska, I Rambabova-Bushljetik, N Gjorgjievski, A Trifunovski, O Ivanovski, V Tasic

**Affiliations:** University Clinic of Nephrology, Skopje, Republic of North Macedonia; University Pediatric Clinic, Skopje, Republic of North Macedonia; University Clinic of Urology, Skopje, Republic of North Macedonia

**Keywords:** Alport syndrome, end–stage kidney disease, kidney transplantation

## Abstract

Alport syndrome is a hereditary disorder caused by a mutation in type IV collagen, leading to progressive kidney disease and eventual end- stage renal failure. Kidney transplantation represents the optimal therapeutic option, typically associated with excellent graft survival. We report a long-term outcome in two brothers with genetically confirmed Alport syndrome (*COL4A5* mutation inherited from their mother, also on hemodialysis). Receiving kidneys from the same deceased donor with only one HLA mismatch. A standard immunosuppression therapy was administered, including Anti-Thymocyte Globulin (ATG) as an induction, followed by maintenance therapy (steroids, a calcineurin inhibitor, and mycophenolic acid). Graft function in both recipients was established immediately, with diuresis up to 8 L and normalized serum creatinine within the first 24 hours after transplantation. During 12 years of follow-up, graft function remained stable, with normal serum creatinine levels, no occurrence of proteinuria, and no development of anti-HLA antibodies. The younger brother developed gingival hyperplasia and steroid-induced diabetes as complications of the therapy. Both patients remain employed and have families.

## INTRODUCTION

Alport syndrome is an inherited disorder caused by pathogenic variants in the *COL4A3, COL4A4*, and *COL4A5* genes encoding type IV collagen, a major structural component of the glomerular basement membrane [1,2]. The disease is characterized by progressive nephropathy, sensorineural hearing loss, and ocular abnormalities, frequently leading to end-stage kidney disease (ESKD) at a young age [1,2]. Kidney transplantation is considered the optimal treatment for patients with ESKD due to Alport syndrome and is associated with excellent patient and graft survival [2]. Unlike many other primary kidney diseases, recurrence of the original disease in the renal allograft is exceptionally rare, contributing to favorable long-term outcomes [3,4,5]. Although several registry studies have reported excellent transplant results in Alport syndrome, reports describing transplantation of kidneys from a single deceased donor into two siblings with genetically confirmed disease remain scarce [4,5]. We present a unique case of two brothers with X-linked Alport syndrome who achieved excellent graft function and complete social rehabilitation more than 10 years after transplantation.

### Case 1

The older brother was hospitalized at the UC of nephrology at the age of 27. Focal segmental glomerulosclerosis was established by kidney biopsy. Genetic tests confirmed the mutation in *COL4A5*, ex49, c.4743_4744delAG, p.Met1582fs, and the diagnosis of Alport syndrome. Hypertension and hearing impairment, but not vision impairment, were present at the time of diagnosis. Fast progression to end-stage chronic kidney disease was observed, and patient undergo to hemodialysis for 9 months. In 2014, he received a kidney from DCD at the age of 40, with no comorbidities. The immunosuppressive regimen consists of induction therapy with Anti - thymocyte globulin (ATG) and triple maintenance therapy that includes steroids, mycophenolic acid (MMF), and calcineurin inhibitor-tacrolimus. The graft function was established in the first 24 hours, with diuresis of 8 L and normalization of serum creatinine. During the follow-up, no episodes of rejection were observed. On graft biopsy 12 months after transplantation, no elements for glomerulonephritis were observed, and BANFF classification was g1, i1, t1, a0, ah0, cg0, cv0, ct0, ca0. One episode of COVID-19, and the operation of cataract were noted. The graft function was stable for the next 10 years, and no DSA was observed. ( Figure. 1 and Table 1). The patient is working and got daughter, also a carrier of the mutation.

**Figure 1.**
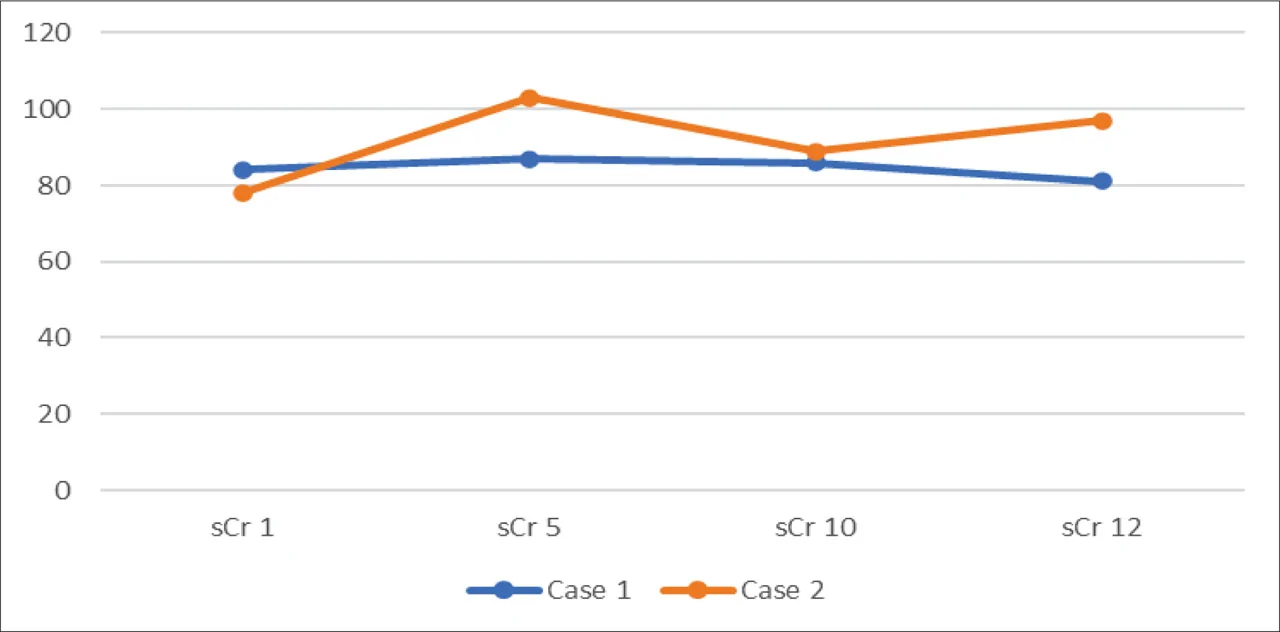
Trend of serum creatinine during the 12 years follow up (sCr/µmol/L/referent values 45–109 /µmol/L)

**Table 1.** Trend of proteinuria during the 12 years follow up.

**Proteinuria g/L**	**1 year**	**5 years**	**10 years**	**12 years**
**Case 1**	0,01	0,02	0,09	0,07
**Case 2**	0,01	0,04	0,06	0,02

### Case 2

The younger brother was admitted at the UC of nephrology with hypertensive crisis and end-stage kidney disease at the age of 26. He was on CAPD for 22 months. Hypertension, hearing impairment, and one episode of cerebrovascular event, with no sequela, were present. In 2014, the kidney transplantation was performed from the same cadaver. The identical, quadruple immunosuppressive protocol was applied. The graft function was established in the first 24 hours, with diuresis of 8 L and normalization of serum creatinine. During the follow-up, no episodes of rejection were observed. On graft biopsy 12 months after transplantation, no elements for glomerulonephritis were observed, and BANFF classification was g1, i0, t1, a0, ah0, cg0, cv0, ct0, ca0. Steroid diabetes and vision impairment occurred during the follow-up. The graft function was stable for the next 10 years, and no DSA was observed (Figure1). The patient returned to work in the first 7 years and got sun, health, but not yet tested.

## DISCUSSION

Kidney transplantation remains the treatment of choice for patients with ESKD secondary to Alport syndrome. Long-term registry data have consistently demonstrated patient and graft survival rates that are comparable to or superior to those observed in recipients transplanted for other causes of kidney failure [ 2, 3, 4, 5]. This is also confirmed in the data from the New Zealand Dialysis and Transplant Registry (ANZDATA) and in the ERA Registry [6,7]. Both conclude that renal transplantation is a major option for patients with Alport Syndrome. Because of genetic structure and possible influence, the authors to use the decease donor transplantation instead of living. Regarding the possible post-transplant anti-GBM nephritis, which is until now a principal obstacle for using transplantation in recipients with Alport Syndrome, the real percentage of such complications is only 0.4% of all recipients. The crucial point is to choose a suitable donor for transplantation. Similar results are reported from the Irish Transplant registry, where in 51 cases with Alport syndrome, 20-year patients and 46.8 graft survival are confirmed, which is numerically superior to outcomes observed in recipients with other primary kidney disease. Genotype-specific studies also demonstrated excellent transplant outcomes. In European countries 10-year graft survival rate is near 60%. Importantly, neither severe *COL4A5* mutation nor genetic subtype adversely affected post-transplant outcomes [5,6,7].

The present cases are remarkable for several reasons. Both recipients carried the same *COL4A5* mutation and received kidneys from a single deceased donor with only one HLA mismatch. Immediate graft function was achieved in both patients, with normalization of serum creatinine within 24 hours after transplantation. During more than 10 years of follow-up, both recipients maintained stable renal function, absence of proteinuria, and no detectable anti-HLA antibodies. No episodes of acute rejection or anti-GBM nephritis were observed. These findings compare favorably with the outcomes reported in large registry studies [8,9].

To our knowledge, reports describing two brothers with genetically confirmed X-linked Alport syndrome receiving kidneys from the same deceased donor and maintaining excellent graft function for more than a decade are extremely rare. This case further supports the growing body of evidence demonstrating that kidney transplantation provides outstanding long-term outcomes in patients with Alport syndrome and highlights the potential for durable graft survival even among genetically related recipients sharing the same underlying mutation [8,9].
